# Clinical and in vivo confocal microscopy characteristics of *Candida* keratitis following keratoplasty

**DOI:** 10.1186/s12886-023-03114-w

**Published:** 2023-09-04

**Authors:** Chen Zhang, Fei Li, Hui Liu, Zhe Jia, Shaozhen Zhao

**Affiliations:** Tianjin Key Laboratory of Retinal Functions and Diseases, Tianjin Branch of National Clinical Research Center for Ocular Disease, Eye Institute, School of Optometry, Tianjin Medical University Eye Hospital, No.251, FuKang Road, Nankai District, 300384 Tianjin, China

**Keywords:** *Candida* keratitis, Keratoplasty, Confocal microscopy, Treatment

## Abstract

**Background:**

We present six patients who developed *Candida* keratitis postoperatively. The clinical features, diagnostic testing including in vivo confocal microscopy, and outcomes are presented.

**Methods:**

Six patients who developed *Candida* keratitis following penetrating and endothelial keratoplasty, were referred to Tianjin Medical University Eye Hospital between 2018 to 2021.The diagnosis was established following cultures of either corneal scraping or biopsy. In vivo confocal microscopy examination was also performed to confirm the diagnosis and characterize the morphology, distribution and the depth of *Candida spp.* All patients were treated with topical voriconazole (VCZ) 1% and natamycin (NTM) 5%. Patients with mid/deep stromal keratitis or interface infection were treated additionally with intrastromal or interface VCZ irrigation (0.05 mg/0.1mL).

**Results:**

The cultures of corneal scrapings (4 cases) or biopsies (2 cases) were all positive for *Candida spp*. In vivo confocal microscopy examination was positive for fungal elements in five of the six patients. The infection resolved in five of the six patients. The patients’ final uncorrected visual acuity (UCVA) ranged from hand movements (HM) to 20/80.

**Conclusion:**

In vivo confocal microscopy is a useful non-invasive clinical technique for confirming the diagnosis of *Candida* keratitis. Intrastromal and interface irrigated VCZ injections are effective treatment options.

**Supplementary Information:**

The online version contains supplementary material available at 10.1186/s12886-023-03114-w.

## Background

Corneal transplantation is the most commonly performed tissue or organ transplant worldwide. Penetrating keratoplasty (PKP), a procedure consisting of full-thickness replacement of the cornea, has been the dominant procedure for more than half a century. In recent years, lamellar keratoplasty techniques have replaced PKP for anterior and posterior corneal pathologies. These include deep anterior lamellar keratoplasty (DALK), Descemet stripping automated endothelial keratoplasty (DSAEK)/Descemet membrane endothelial keratoplasty (DMEK).

Post-keratoplasty infectious keratitis is a devastating complication that may severely affect corneal graft survival rate and visual outcomes [1]. Fungal keratitis post-keratoplasty is actually more common than bacterial or viral keratitis [2, 3]. A study by the Eye Bank Association of America encompassing 4 years (2007 − 2010) of activity, reported a cumulative frequency of post-keratoplasty infection rate of 0.026% for fungal and bacterial agents together, with a higher rate for fungal isolates (63%). The most common fungal infection, particularly in endothelial keratoplasty (EK), are from the *Candida* genus, with the majority caused by either *Candida albicans* or *Candida parapsilosis* [4].

Early-onset *Candida* infection after keratoplasty commonly presents as a white infiltrate that may not be associated with significant inflammation. The dense infiltration can result in vision loss especially in patients with deep interface infection after EK, which can also spread to the anterior chamber resulting in endophthalmitis. Early diagnosis of post-keratoplasty *Candida* infection is very important for prognosis. Confocal microscopy is a useful tool to confirm the diagnosis of the infectious microorganism especially in patients with deep corneal infections. It has a good diagnostic accuracy especially for amoebic keratitis and fungal keratitis [5, 6]. Treatment of fungal keratitis is a clinical challenge. Intrastromal and interface antifungal therapy has been effective in arresting the progression of fungal infection for late-onset lamellar interface infectious keratitis [7].

In this study, we report six cases of *Candida* keratitis after keratoplasty (3 post PKP and 3 following DSAEK). We documented the clinical and confocal microscopy features, as well as the outcomes of the management.

## Methods

This study was performed in accordance with the Declaration of Helsinki. The study was approved by the ethics committee of Tianjin Medical University Eye Hospital. Written informed consent was obtained from the patients. Six patients with laboratory diagnosis of *Candida* keratitis after keratoplasty were examined at Tianjin Medical University Eye Hospital from 2018 to 2021. Culture of corneal scraping (4 cases) and corneal biopsy (2 cases) were performed to establish the diagnosis.

Specimens for corneal scraping cytological analysis were obtained using a surgical blade and the sample was fixed in methanol, then stained with Giemsa. Specimens for culture were also obtained using a surgical blade. In 2 post-DSAEK patients with interface infection, a corneal biopsy was taken in the operating theater under topical anesthesia. A corneal incision at the peripheral edge of the infiltrate was performed, taking care to avoid the visual axis. Specimens from corneal scrapings or a tissue biopsy were inoculated on Sabouraud dextrose agar (at 22–25 °C) and blood agar (at 37 °C). Isolates were identified by MALDI-TOF-MS system (Bruker, Germany).

In vivo confocal microscopy scanning with the HRTIII-RCM (Heidelberg Engineering GmbH, Dossenheim, Germany) was performed on all patients. The morphology, distribution and depth of *Candida spp.* in the cornea was observed using 800x magnification.

All patients were immediately treated with topical voriconazole (VCZ) 1% and topical natamycin (NTM) 5% every hour, when the result of in vivo confocal microscopy examination or corneal scraping for cytological analysis was positive. Patients with mid/deep stromal keratitis or interface infection were treated with a combination of oral VCZ (400 mg, two times a day). Two post-PKP patients with mid/deep stromal keratitis were additionally treated with intrastromal VCZ injection. Intrastromal injection of VCZ (0.05 mg/0.1mL) with a 30-g needle around the active peripheral lesion was performed. The amount of drug injected intrastromally was about 0.1mL. And two post-DSAEK patients with interface infection were additionally treated with interface VCZ irrigation. Interface irrigation with VCZ (0.05 mg/0.1mL) was also performed with a 30-g needle. The amount of drug was about 0.1mL.

## Results

The clinical data of the patients in this study was outlined in Table 1. The 6 patients included three males and three females, between 32 and 89 years old. Three patients underwent PKP and three underwent DSAEK. After surgery, all the patients were given topical corticosteroids to reduce the risk of graft rejection. The time to onset of infection after surgery ranged from 1 to 9 months. The cytological analysis of the corneal scrapings was positive for *Candida* in four of six patients. The culture of the corneal scraping (4 cases)/ biopsy (2 cases) for *Candida spp.* was positive in all patients. *Candida albicans* was the most common species isolated (4 cases), followed by *Candida dubliniensis* (1 case) and *Candida lusitaniae* (1 case).

**Table 1:** Clinical data of patients with *Candida* keratitis after keratoplasty

Patient No., age, gender	Eye	Type of surgery	Interval to onset of infection	Clinical manifestation	Corneal scraping cytological analysis	Corneal scraping/biopsy culture	Confocal microscopy	Treatment	Time of treatment	UCVA	
										Before treatment	After treatment
1,89,M	OS	DSAEK	5 months	White infiltrate in the mid anterior stroma	+	Candida dubliniensis	+	VCZ (topical) NTM (topical)	1 month	CF	20/200
2,65,M	OS	PKP	3 months	White infiltrate in the mid stroma	+	Candida albicans	+	VCZ (topical + intrastromal) NTM (topical)	45 days	20/160	20/80
3, 56,M	OD	DSAEK	2 months	Graft-host interface infiltration	-	Candida albicans	+	VCZ (topical + interface irrigation + oral) NTM (topical)	2 months	20/160	20/80
4,62 F	OS	DSAEK	1 month	Graft-host interface infiltration	-	Candida albicans	-	VCZ (topical + interface irrigation + oral) NTM (topical) Remove graft	2 months	CF	HM
5,32,F	OD	PKP	3 months	White infiltrate in the deep stroma around the loose suture	+	Candida albicans	+	VCZ (topical + intrastromal + oral) NTM (topical)	2 months	20/100	20/80
6, 67,F	OS	PKP	9 months	White infiltrate in the anterior stroma	+	Candida lusitaniae	+	VCZ (topical) NTM (topical)	2 months	CF	20/200

Abbreviations: PKP, penetrating keratoplasty; DSAEK, Descemet stripping automated endothelial keratoplasty; VCZ, voriconazole; NTM, natamycin; UCVA, uncorrected visual acuity; CF, counting fingers; HM, hand movements; M, male; F, female; No, number

In vivo confocal microscopy examination was positive in five of six patients (3 post-PKP and 2 post-DSAEK). Fungal spores were identified in all five patients. One patient was positive for both spores and pseudohyphae structures. The fungal spores were hyperreflective round deposits, about 2–5 μm in diameter, distributed in clusters. No obvious inflammatory cell infiltration was observed around the fungal spores. Pseudohyphae were characterized by high-density striate structures with clusters of round spore images around them.

After therapy, infection in five of six patients was completely resolved within 45–60 days. Only one post-DSAEK patient (case 4) with severe infection had the corneal endothelium graft removed. Final uncorrected visual acuity (UCVA) ranged from HM to 20/80.

## Case 1

**Fig. 1:**
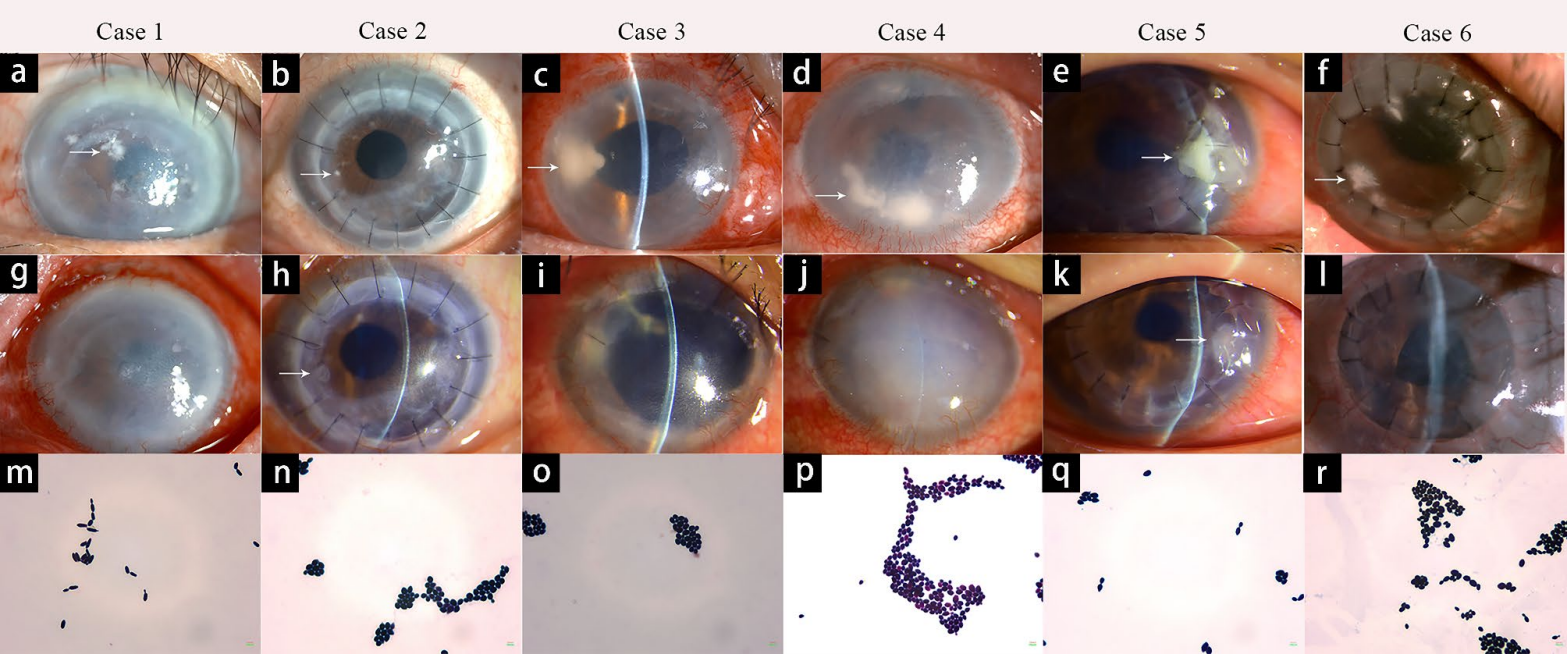
Clinical features and culture results of six patients. **Case 1**, a post-DSAEK patient. Slit-lamp examination showing multifocal white infiltrates in the central anterior stroma with an overlying corneal epithelial defect (**a**). The corneal infiltrate resolved and the epithelium was healing (**g**). Gram stain of scraping demonstrating *Candida dubliniensis* (Gram staining, 1000×) (**m**). **Case 2**, a post-PKP patient. Slit-lamp examination showing small, round white infiltrate in the peripheral graft (**b**). The corneal scar formed after treatment (**h**). Gram stain of scraping demonstrating *Candida albicans* by culture (Gram staining, 1000×) (**n**). **Case 3**, a post-DSAEK patient. Slit-lamp examination demonstrating a dense infiltrate in the interface at the temporal edge of the corneal endothelial graft (**c**). The infiltrate gradually resolved and the central cornea remained clear (**i**). The result of culture was *Candida albicans*, seen with Gram stain (1000×) (**o**). **Case 4**, a post-DSAEK patient. Slit lamp examination revealed large (3 × 6 mm) infiltrate at the inferior graft-host interface (**d**). The endothelial graft was removed with resultant severe corneal edema (**j**). The result of the culture was *Candida albicans*, seen with Gram stain(1000×) (**p**). **Case 5**, a post-PKP patient. The slit-lamp examination showed a large (about 2 × 2 mm), dense infiltrate in the peripheral graft surrounding a loose suture (**e**). The corneal infiltrate gradually resolved following 15 days of treatment (**k**). The results of the culture was *Candida albicans*, seen with Gram stain, (1000×) (**q**). **Case 6**, a post-PKP patient. Slit-lamp photo of multifocal white infiltrates in the peripheral corneal graft (**f**). After 3 months, the corneal infiltrate resolved and epithelium healed with residual peripheral corneal vascularization (**l**). The result of the culture was *Candida lusitaniae*, seen with Gram stain (1000×) (**r**)

**Fig. 2:**
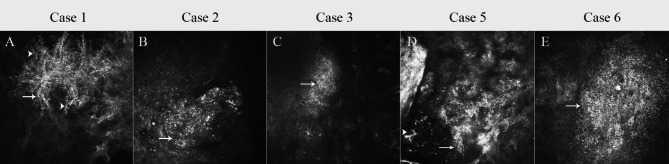
In vivo confocal microscopy images of *Candida* spores and pseudohyphae. The fungal spores (arrows) were hyperreflective round deposits, about 2–5 μm in diameter, distributed in clusters. Pseudohyphae (arrowheads) were mainly characterized by high-density striate structures and clusters of round spore images could be seen around it. (**a**: Case 1, depth: 60 μm; **b**: Case 2, depth: 40 μm; **c**: Case 3, depth: 602 μm; **d**: Case 5, depth: 45 μm; **e**: Case 6, depth: 22 μm)

An 89-year-old male patient underwent uncomplicated DSAEK in the left eye for corneal endothelial decompensation after cataract surgery. Delayed epithelialization lasted for two weeks occurred after DSAEK due to severe dry eye. Five months after surgery, the patient noted a foreign body sensation in the left eye for a few days. The UCVA was counting fingers in the left eye. Slit-lamp examination showed central multifocal white infiltrates in the anterior stroma with an overlying corneal epithelial defect (Fig. 1a). The margin of infiltrates was feathery. The anterior chamber had no cell or flare. Confocal microscopy revealed hyperreflective round deposits, about 2–5 μm in diameter, and distributed in clusters in the anterior stroma. Pseudohyphae structures could also be seen (Fig. 2a). Corneal scraping for cytological analysis and culture were positive for *Candida dubliniensis* (Fig. 1m). Topical VCZ 1% and NTM 5% were started every hour with a gradual taper. After 1 month, the corneal infiltrate resolved and epithelial defect was healing (Fig. 1g) with residual corneal opacity. The final visual acuity was 20/200.

## Case 2

This patient was a 65-year-old male with a history of cerebral ischemia. He underwent PKP for a central corneal leucoma in left eye. At the three-month visit, the patient presented with a central round white infiltrate in the corneal graft (Fig. 1b). The infiltrate was dense without surrounding stromal edema. The patient did not complain of redness or pain. The UCVA was 20/160. Confocal microscopy demonstrated multiple hyperreflective round deposits, about 2 to 5 μm in diameter, distributed in nests in the corneal epithelium (Fig. 2b). The corneal scraping demonstrated was *Candida albicans* on culture (Fig. 1n). The patient was started on topical VCZ 1% and NTM 5% every hour as well as an intrastromal injection of VCZ (0.05 mg/0.1mL) around the infiltrate using a 30-g needle. Forty-five days later, the infiltrate resolved leaving a residual corneal scar (Fig. 1h). The UCVA was 20/80.

## Case 3

**Fig. 3:**
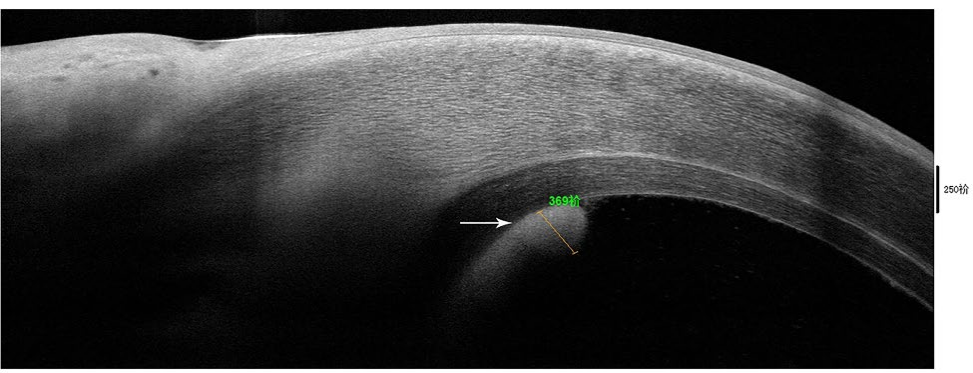
Anterior segment optical coherence tomography (AS-OCT) examination of case 3. AS-OCT showed dense infiltrate in the endothelium

A 56-year-old male patient underwent uncomplicated DSAEK in the right eye for corneal endothelial decompensation after unsuccessful cataract surgery. At the two-month visit, the patient complained of pain and redness in the right eye. The UCVA in the right eye was 20/160. Slit-lamp exam and anterior segment optical coherence tomography (AS-OCT) demonstrated a dense infiltrate in the interface and endothelium at the temporal edge of the endothelial graft, about 3 × 4 mm, posteriorly toward the anterior chamber (Figs. 1c and 3). Small hyperreflective granular deposits measuring 2 to 5 μm in diameter were identified at the interface by confocal microscopy (Fig. 2c). A corneal biopsy was obtained and *Candida albicans* infection was verified by the culture (Fig. 1o). Antifungal therapy was started with topical VCZ 1% and NTM 5% every hour with a gradual taper. In addition, the patient received interface irrigation with VCZ (0.05 mg/0.1mL) using a 30 g needle for three times on day 1, day 7 and day 14. Oral VCZ (400 mg, two times a day) was also given for two weeks. Two months later, the infiltrate gradually resolved leaving a peripheral stromal scar. The center of corneal endothelial graft was still transparent (Fig. 1i). The UCVA was 20/80 due to irregular astigmatism.

## Case 4

A 62-year-old female patient underwent uncomplicated DSAEK in the left eye for corneal endothelial decompensation following trabeculectomy. After two weeks of the surgery, the UCVA was 20/200 due to a long history of glaucoma. At one month, the patient presented with a decrease in visual acuity and redness. On examination, the UCVA was counting fingers. Slit-lamp examination revealed a 3 × 6 mm white infiltrate and another superior infiltrate in the graft-host interface and without any overlying epithelial defect or hypopyon (Fig. 1d). However, the confocal microscopy interface images were not clear due to severe stromal edema. Under confocal microscopy, fungal spores or hyphae were not found. Because the infiltrate was internal, we performed a biopsy of the infiltrate which demonstrated *Candida albicans* infection (Fig. 1p). The patient was started on topical VCZ 1% and NTM 5% every 1 h, combined with interface irrigation with VCZ (0.05 mg/0.1mL) two times. Oral VCZ (400 mg, two times a day) was also given. The graft dislocated during the third interface irrigation, so we removed it. After 2 months of therapy, the infiltrate resolved with residual severe corneal edema. And she did not develop endophthalmitis (Fig. 1j). The patient was scheduled for a PKP.

## Case 5

A 32-year-old female patient underwent PKP in the right eye because of acute corneal hydrops associated with keratoconus. After 1 week of the surgery, the UCVA was 20/80. But at the three-month visit, the patient presented with complaints of pain and redness in the right eye for 3 days. Her UCVA decreased to 20/100. Slit-lamp examination showed a dense 2 × 2 mm peripheral corneal infiltrate around a loose suture (Fig. 1e). Small hyperreflective granular deposits measuring 2 to 5 μm in diameter were found on confocal microscopy (Fig. 2d). The culture of the corneal scraping demonstrated *Candida albicans* (Fig. 1q). Topical VCZ 1% and NTM 5% were started every 1 h. The patient also received 2 injections of intrastromal VCZ (0.05 mg/0.1mL). Oral VCZ (400 mg, two times a day) was also started. The infiltrate gradually resolved after 15 days (Fig. 1k) and the antifungal topical drops were tapered. After 2 months, the UCVA in the right eye was 20/80.

## Case 6

The patient was a 67-year-old female patient with a history of Stevens-Johnson Syndrome (SJS) at the age of 47. She had severe corneal vascularization and opacification, conjunctival fibrosis, and symblepharon formation in both eyes. She underwent oral mucosal epithelial transplantation, amniotic membrane transplantation combined with limbal stem cell transplantation in the left eye. After 1 year, PKP was performed because of corneal opacity and vascularizaton. The postoperative course was complicated by delayed epithelialization. At the two-month visit, the patient presented with a multifocal white infiltrate in the peripheral corneal graft (Fig. 1f). Her UCVA was counting fingers. Confocal microscopy revealed hyperreflective round deposits, about 2–5 μm in diameter, in the anterior stroma. (Fig. 2e). The culture of corneal scraping was positive for *Candida lusitaniae* (Fig. 1r). Topical VCZ 1% and NTM 5% was started every 1 h. After 3 months, the corneal infiltrate resolved and epithelial defect resolved as well, with residual mild edema graft and peripheral corneal (stromal and or superficial) neovascularization. (Fig. 1l). The UCVA was 20/200.

## Discussion

Post-keratoplasty infectious keratitis is a well-known clinical challenge. The incidence of infectious keratitis after keratoplasty varies widely, ranging from 0.02 to 11.9% depending on the specific type of transplant (PKP, DALK, or EK) [8–10]. Early diagnosis and treatment of post-keratoplasty *Candida* infection is important, especially in cases of deep stromal or interface infiltrates. In this study, six patients who developed *Candida* keratitis postoperatively were collected. The clinical features, diagnostic testing including in vivo confocal microscopy, and treatment outcomes were analyzed.

Topical corticosteroids are usually administered following keratoplasty to reduce the risk of graft rejection [8]. The use of corticosteroids, however, is a double-edged sword. A 15-year review of clinical outcomes of 21 cases of *Candida* keratitis in a Canadian eye center suggested that the use of topical corticosteroids was a common risk factor [11]. In our study, topical steroids were routinely given to all patients following keratoplasty. The local immunosuppressive effect of steroids may be an important risk factor for *Candida* infection. Other well-known risk factors for infectious keratitis include persistent epithelial defects and loose sutures [12]. Two of our patients (one post-PKP, the other post-DSAEK) had a history of delayed epithelialization. In our series of patients, severe ocular surface disease (SJS) and tear dysfunction were also contributing risk factors. One of our post-PKP patients (case 5) had a loose suture, a well-known risk factor. Loose sutures cause epithelial defects which can be contaminated by environmental and ocular surface commensals.

Post keratoplasty *Candida* keratitis is typically asymptomatic associated with minimal inflammation. The patients may present with a quiet white eye, with mild vision loss as the only presenting symptom, especially in early-onset interface infection after DSAEK. Most *Candida* interface keratitis develop several weeks to several months after EK surgery [13]. In our series, the interval from surgery to onset of infection was 1 to 9 months. Since the sequestered location of the infection in the deep stroma or interface along with the reduced virulence of the *Candida* organism, is associated with an asymptomatic presentation, the diagnosis is often delayed. Furthermore, the impaired access to the deep location of the organism, contributes to the challenges of microbiological testing and the clinical management of these cases.

In this restrictive setting of cases of *Candida spp.* infection, in vivo confocal microscopy can provide an important diagnostic tool by identifying hyperreflective round structures consistent with *Candida*. In our series, confocal microscopy was positive in five of six patients. Our imaging of *Candida* spores was similar to previous reports [14, 15]. The size of the spores was about 2–5 μm in diameter, distributed in clusters. Pseudohyphae were identified by their characteristic high-density striate structure. We also noted a lack of inflammatory cell infiltration around spores and pseudohyphae. Three different species of *Candida* were identified in our patients. Although the size and appearance of these 3 species of spores were similar, the pseudohyphae were only found in the patient with *Candida dubliniensis* keratitis (case 1). In vitro formation of pseudohyphae is related to the species of *Candida*, the type of culture plates and the temperature. Pseudohyphae is also an indication of the virulence of the *Candida spp*. In the identification test between *Candida albicans* and *Candida dubliniensis* on staib agar, *Candida dubliniensis* produced rough colonies, peudohyphae and chlamydospores. Although the *Candida albicans* produced pseudohyphae after prolonged growth [16].

However, in our opinion, we were unable to accurately identify the genus of *Candida* from in vivo confocal microscope images. Morphological features of fungal colonies and spores require up to 7 days for fungi to grow and sporulate in culture. In contrast, the high-resolution imaging modality of in vivo confocal microscopy provides immediate visualisation of fungi within living cornea.

In general, cultures of corneal scraping constitute the standard methodology to diagnose fungal infection [17]. However, in our study, due to the deep location of the infection in two post-DSAEK patients who had developed graft-host interface infections, corneal scrapings would not be helpful in identifying the culprit organism. For these 2 cases, we performed interface corneal biopsy. Firstly, a corneal incision at the peripheral edge of the infiltrate was performed. Then we use the forceps to get the specimens. The results showed *Candida albicans* infection. Corneal biopsy is the definitive procedure to establish a diagnosis in progressive keratitis, especially if corneal scraping yields negative results [18]. Notwithstanding this technique carries some risks, including intraoperative corneal perforation as well as corneal scarring.

Standard topical antifungal therapy with NTM 5% and VCZ 1%, has limited efficacy for deeper interface infection due to the poor penetration [19]. Intrastromal injection and interface irrigation of VCZ is a preferred adjunctive treatment approach in the management of deep *Candida* keratitis. This therapy can provide maximum drug load at the site of the infection, especially at the graft–host interface, with the aim of salvaging the graft and avoiding PKP [20]. In a previous study, the success rate of intrastromal VCZ in treating recalcitrant fungal keratitis (ulcer size > 2 mm, a depth > 50% of stroma, and not responding to topical NTM therapy for two weeks) was 95% [21]. In addition, interface irrigation with antifungal agents has reportedly been effective in clearing interface infection, but carries a risk of graft dislocation [9]. This adjunctive treatment was effective in our patients, in which the graft was successfully salvaged. In case 4, however, with severe interface infection after DSAEK, the graft was dislocated when we performed interface irrigation. Therefore, we had to remove the graft and plan for a PKP. One of the main limitations of our study was the small sample size. Other limitations included a lack of culture results for donor cornea rim. So the risk of the contamination from the donor tissue could not be analyzed. Occurrence of fungal keratitis in the recipient of the donor’s fellow eye may be indicative of donor related source of infection. Of course, during the surgery of fungal keratitis, we should notice sufficient graft diameter, always interrupted sutures, no double running suture, and anterior chamber irrigation.

In conclusion, *Candida spp.* demonstrates characteristic morphological features on in vivo confocal microscopy. In cases of deep stromal or interface infiltrates following keratoplasty, which may be inaccessible by corneal scraping, the use of confocal microscopy demonstrating white deposits with hyperreflective round structures, may provide important support of the possibility of *Candida* keratitis. In these cases, intrastromal and interface injection of antifungal agents can be a safe and useful adjunct to standard topical and systemic antifungal therapy for the management of fungal keratitis. Early diagnosis and treatment may lead to a better prognosis for patients with *Candida* keratitis after keratoplasty.

### Electronic supplementary material

Below is the link to the electronic supplementary material.

Supplementary Material 1

## Data Availability

All data generated or analyzed during this study are included in this published article.
