# Supplementary material for: Clinical and in vivo confocal microscopy characteristics of Candida keratitis following keratoplasty
Source: BMC Ophthalmol. 2023 Sep 4;23:364. doi: 10.1186/s12886-023-03114-w (PMC10476318; doi:10.1186/s12886-023-03114-w)
Supplement: Supplementary file 1 — Supplementary Material 1 [file 12886_2023_3114_MOESM1_ESM.pdf]

Consent for Publication of Identifying Material in BMC Opthalmology

I give my permission for the following material to appear in the print, online, and licensed versions of BMC Ophthalmology Journal and for the Journal to grant permission to third parties to reproduce this material.

Title or subject of article or photograph, video, or audio: Clinical and in vivo confocal microscopy characteristics of *Candida* keratitis following keratoplasty

I understand that my name will not be published but that complete anonymity cannot be guaranteed.

I have read the manuscript or a general description of what the manuscript contains and reviewed all photographs, illustrations, video, or audio files (if included) in which I am included that will be published.

Liqing Wang ______ 19 July 2023 ______

Signed Date

Liqing Wang ______

Print name

______

If you are granting permission for another person, what is your relationship to that person?

Consent for Publication of Identifying Material in BMC Opthalmology

I give my permission for the following material to appear in the print, online, and licensed versions of BMC Ophthalmology Journal and for the Journal to grant permission to third parties to reproduce this material.

Title or subject of article or photograph, video, or audio: Clinical and in vivo confocal microscopy characteristics of *Candida* keratitis following keratoplasty

I understand that my name will not be published but that complete anonymity cannot be guaranteed.

I have read the manuscript or a general description of what the manuscript contains and reviewed all photographs, illustrations, video, or audio files (if included) in which I am included that will be published.

Guixia Huang ______________ 19 July 2023.

Signed Date

Guixia Huang ______________

Print name

____

If you are granting permission for another person, what is your relationship to that person?

Consent for Publication of Identifying Material in BMC Opthalmology

I give my permission for the following material to appear in the print, online, and licensed versions of BMC Ophthalmology Journal and for the Journal to grant permission to third parties to reproduce this material.

Title or subject of article or photograph, video, or audio: Clinical and in vivo confocal microscopy characteristics of *Candida* keratitis following keratoplasty

I understand that my name will not be published but that complete anonymity cannot be guaranteed.

I have read the manuscript or a general description of what the manuscript contains and reviewed all photographs, illustrations, video, or audio files (if included) in which I am included that will be published.

Deli Zhong ______________ 20 July 2023 ______________

Signed Date

Deli Zhong ______________

Print name

____

If you are granting permission for another person, what is your relationship to that person?

Consent for Publication of Identifying Material in BMC Opthalmology

I give my permission for the following material to appear in the print, online, and licensed versions of BMC Ophthalmology Journal and for the Journal to grant permission to third parties to reproduce this material.

Title or subject of article or photograph, video, or audio: Clinical and in vivo confocal microscopy characteristics of *Candida* keratitis following keratoplasty

I understand that my name will not be published but that complete anonymity cannot be guaranteed.

I have read the manuscript or a general description of what the manuscript contains and reviewed all photographs, illustrations, video, or audio files (if included) in which I am included that will be published.

Yuanyuan Zhou — 20 July 2023

Signed — Date

Yuanyuan Zhou

Print name

If you are granting permission for another person, what is your relationship to that person?

Consent for Publication of Identifying Material in BMC Opthalmology

I give my permission for the following material to appear in the print, online, and licensed versions of BMC Ophthalmology Journal and for the Journal to grant permission to third parties to reproduce this material.

Title or subject of article or photograph, video, or audio: Clinical and in vivo confocal microscopy characteristics of *Candida* keratitis following keratoplasty

I understand that my name will not be published but that complete anonymity cannot be guaranteed.

I have read the manuscript or a general description of what the manuscript contains and reviewed all photographs, illustrations, video, or audio files (if included) in which I am included that will be published.

Daiqiang Liu ______ 20 July 2023 ______

Signed Date

Daiqiang Liu ______

Print name

______

If you are granting permission for another person, what is your relationship to that person?

Consent for Publication of Identifying Material in BMC Opthalmology

I give my permission for the following material to appear in the print, online, and licensed versions of BMC Ophthalmology Journal and for the Journal to grant permission to third parties to reproduce this material.

Title or subject of article or photograph, video, or audio: Clinical and in vivo confocal microscopy characteristics of *Candida* keratitis following keratoplasty

I understand that my name will not be published but that complete anonymity cannot be guaranteed.

I have read the manuscript or a general description of what the manuscript contains and reviewed all photographs, illustrations, video, or audio files (if included) in which I am included that will be published.

Xiuying Wang

Signed

19 July 2023

Date

Xiuying Wang

Print name

If you are granting permission for another person, what is your relationship to that person?
